# Role of SLC7A11/xCT in Ovarian Cancer

**DOI:** 10.3390/ijms25010587

**Published:** 2024-01-02

**Authors:** Sonia Fantone, Federica Piani, Fabiola Olivieri, Maria Rita Rippo, Angelo Sirico, Nicoletta Di Simone, Daniela Marzioni, Giovanni Tossetta

**Affiliations:** 1 Scientific Direction, IRCCS INRCA, 60124 Ancona, Italy; s.fantone@inrca.it (S.F.); f.olivieri@staff.univpm.it (F.O.); 2 Hypertension and Cardiovascular Risk Research Center, Medical and Surgical Sciences Department, Alma Mater Studiorum University of Bologna, 40126 Bologna, Italy; federica.piani@unibo.it; 3 Department of Clinical and Molecular Sciences, DISCLIMO, Università Politecnica delle Marche, 60126 Ancona, Italy; m.r.rippo@staff.univpm.it; 4 Obstetrics and Gynecology Unit, Sant’Anna e San Sebastiano Hospital, 81100 Caserta, Italy; siricoangelo@gmail.com; 5 Department of Biomedical Sciences, Humanitas University, 20072 Milan, Italy; nicoletta.disimone@hunimed.eu; 6 IRCCS Humanitas Research Hospital, 20089 Rozzano, Italy; 7 Department of Experimental and Clinical Medicine, Università Politecnica delle Marche, 60126 Ancona, Italy; d.marzioni@staff.univpm.it

**Keywords:** SLC7A11, ovarian cancer, chemoresistance, nuclear factor erythroid 2-related factor 2 (NRF2), oxidative stress, reactive oxygen species (ROS), non-coding RNAs, p53, compounds, xCT

## Abstract

Ovarian cancer is one of the most dangerous gynecologic cancers worldwide and has a high fatality rate due to diagnosis at an advanced stage of the disease as well as a high recurrence rate due to the occurrence of chemotherapy resistance. In fact, chemoresistance weakens the therapeutic effects, worsening the outcome of this pathology. Solute Carrier Family 7 Member 11 (SLC7A11, also known as xCT) is the functional subunit of the $X_{c}^{-}$ system, an anionic L-cystine/L-glutamate antiporter expressed on the cell surface. SLC7A11 expression is significantly upregulated in several types of cancers in which it can inhibit ferroptosis and favor cancer cell proliferation, invasion and chemoresistance. SLC7A11 expression is also increased in ovarian cancer tissues, suggesting a possible role of this protein as a therapeutic target. In this review, we provide an overview of the current literature regarding the role of SLC7A11 in ovarian cancer to provide new insights on SLC7A11 modulation and evaluate the potential role of SLC7A11 as a therapeutic target.

## 1. Introduction

Gynecologic cancers are common among older women. Almost 50% of ovarian cancers occur in women aged >65 years and are the major cause of death since most diagnoses are often made when the disease is already at an advanced stage [1]. Although the most common risk factors for developing ovarian cancer are genetic and epigenetic factors, environmental factors and lifestyle also have important roles in its etiopathogenesis [2,3].

In fact, the depletion of ovarian function during aging not only leads to a reduced reproductive capacity but also increases the risk of ovarian cancer. This cancer can therefore be considered an aging-related disease [4]. 

Ovarian cancer can be subdivided into three types: epithelial ovarian cancer (EOC, representing most ovarian cancers diagnosed), ovarian germ cell tumors and stromal cell ovarian tumors. EOC can be further subdivided into four different histological subtypes: serous (the most common), endometrioid, clear cell and mucinous [5,6].

Surgery followed by platinum/taxane chemotherapy is the current treatment for advanced ovarian cancer, but the survival rate is very low, and many patients relapse within 18 months due the occurrence of chemoresistance [5].

Platinum-derived drugs (cisplatin, carboplatin and oxaliplatin) interact with DNA to form mono-adducts and intra- and inter-strand crosslinks that block DNA synthesis and transcription, leading to cell death [7]. In addition, treatment with platinum-derived drugs induces the formation of reactive oxygen species (ROS), leading to DNA damage and apoptosis [8]. Due to this latter mechanism of action of platinum-derived drugs, the glutathione (GSH) content of the cells plays a key role in drug efficiency since GSH can efficiently inactivate the increased ROS induced by platinum-derived drugs [8]. 

Taxanes (e.g., Paclitaxel) exert their anticancer effects by preventing tubulin depolymerization. This process inhibits microtubule shortening during anaphase, which blocks sister chromatid separation, leading to cell death [9]. One of the most common mechanisms of taxane resistance, which is also involved in platinum-based chemotherapies, is the pumping out of the drug by efflux transporters, such as the ATP-binding cassette transporter (known as ABCB1, P-glycoprotein, P-gp or MDR1) [10,11]. In addition to this mechanism, the anticancer effects of paclitaxel can be counteracted by the activation of pro-mitotic factors, such as the phosphoinositide 3-kinase/protein kinase B (PI3K/AKT) pathway [12], and an increase in the expression of the anti-apoptotic members of B-cell lymphoma 2 (Bcl-2) family [13,14].

Finding novel diagnostic biomarkers and therapeutic targets is a major challenge in oncology [15,16]. Thus, identifying new biomarkers in ovarian cancer is urgently required due to the high aggressiveness of this neoplasm in order to improve the outcome of this disease by avoiding/delaying the onset of chemoresistance. 

Oxidative stress is characteristic of the cancer cell microenvironment, and cancer cells protect themselves by increasing their antioxidant defenses [17,18,19,20]. Cysteine can be involved in maintaining redox homeostasis since it is used by the cells to synthesize glutathione (GSH) [21,22]. Intracellular cysteine can be produced by recycling it from degraded proteins or by de novo synthesis [23]. In tumor cells, neither the recycling of proteins to produce cysteine nor its de novo synthesis is sufficient; therefore, cancer cells rely on nutrient transporters to import cystine (the oxidized form of cysteine) from the extracellular microenvironment [23,24,25]. 

Solute Carrier Family 7 Member 11 (SLC7A11, also known as xCT) is the functional subunit of the $X_{c}^{-}$ system, an anionic L-cystine/L-glutamate antiporter expressed on the cell surface. The SLC7A11 requires that solute carrier family 3 member 2 (SLC3A2) import extracellular cystine in exchange for intracellular glutamate at a 1:1 molar ratio [26,27]. Although SLC3A2 can serve as a chaperone protein for other amino acid transporter systems, SLC7A11 is specific for the $X_{c}^{-}$ system [26,27]. SLC7A11 imports extracellular oxidized cystine into the cell, and it is then converted to cysteine [23,28]. The subsequent synthesis of GSH reduces intracellular ROS levels, thereby blocking lipid peroxidation and ferroptosis [29]. 

**Figure 1:**
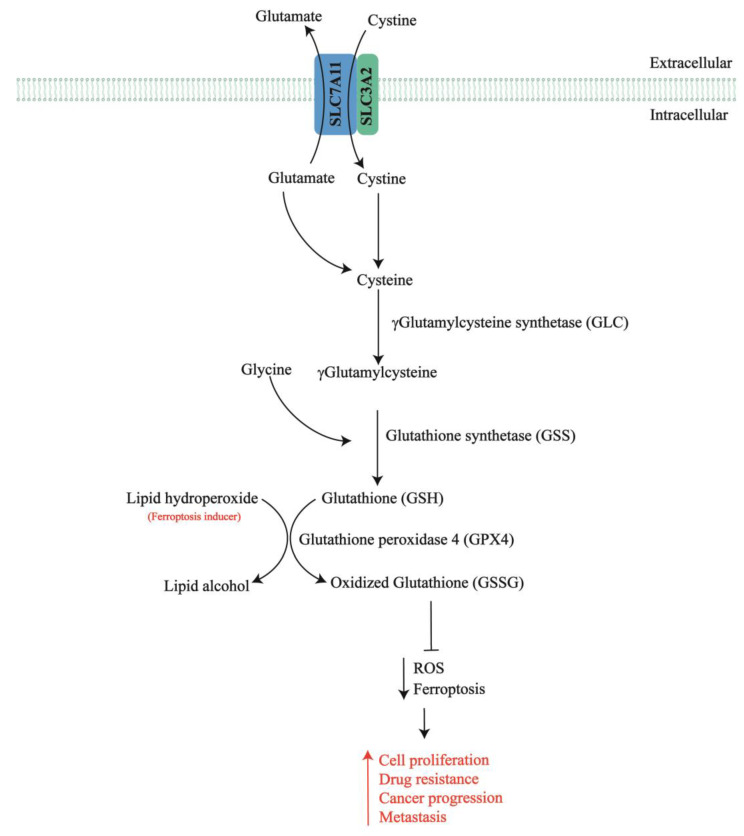
Representation of the function of SLC7A11. SLC7A11 imports extracellular cystine, which is reduced to cysteine and is used in conjugation with glutamate to form γglutamylcysteine (reaction catalyzed by GLC). γGlutamylcysteine is conjugated with glycine (via GSS) to produce GSH, which is used as a cofactor for enzymes, such as GPX4, that protect cells from lipid peroxidation. In particular, GPX4 uses GSH to reduce lipid hydroperoxides (a ferroptosis inducer) to lipid alcohols, suppressing ferroptosis. In this reaction GSH is oxidized to GSSG. GSH also protects cells from ROS exposure by decreasing ROS levels. Thus, high SLC7A11 levels in cancer cells cause cell proliferation, drug resistance, cancer progression and metastasis.

A schematic representation of the function of SLC7A11 is shown in Figure 1. 

Since SLC7A11 expression is upregulated in several types of human cancers, it could be an important therapeutic target for cancer treatment [23,28,30].

The aim of this review is to provide an overview of the current literature regarding the role of SLC7A11 in ovarian cancer in order to provide new insights into SLC7A11 modulation and to evaluate the potential role of SLC7A11 as a therapeutic target.

## 2. SLC7A11 in Chemotherapy-Resistant Ovarian Cancer

Qin and colleagues reported that SLC7A11 protein expression was significantly increased in ovarian cancer tissues and cisplatin-resistant A2780/DDP and SKOV3/DDP cells compared to the cisplatin-sensitive cells [31]. These data are in disagreement with those reported by Yin and colleagues who reported a significant downregulation of SLC7A11 mRNA in platinum-resistant tissues compared to the platinum-sensitive ones. Authors also found that *SLC7A11* gene expression was an independent prognostic factor for overall survival [32].

Thus, although SLC7A11 could be a potential therapeutic target in ovarian cancer treatment, more studies are required to evaluate the role of SLC7A11 expression as a biomarker in ovarian cancer management. 

Another study found that patients with epithelial ovarian cancer (EOC) and a high co-expression level of SLC7A11 and GPX4 had a 60-fold higher risk of developing platinum resistance compared with patients with a low level of co-expression. In addition, a high co-expression level of SLC7A11 and GPX4 was an independent prognostic factor of poor overall survival and poor progression-free survival. Moreover, expression levels of both SLC7A11 and GPX4 were upregulated in platinum-resistant A2780/DDP and SKOV3/DDP cells compared with the platinum-sensitive A2780 and SKOV3 parental ovarian cancer cells. Interestingly, the silencing of SLC7A11 or GPX4 significantly decreased platinum resistance. Thus, high expression levels of SLC7A11 and GPX4 are associated with platinum resistance in EOC patients and may be significant independent prognostic factors and potential therapeutic targets in EOC patients with platinum resistance [33].

A combined platinum and paclitaxel chemotherapy regimen after surgery is a first-line treatment for ovarian cancer patients, but resistance frequently occurs, causing chemotherapy failure [34]. Competing endogenous RNAs (ceRNAs) are RNA molecules that can bind to miRNA response elements (MREs) located on the 3′UTRs of mRNAs and can thus compete with other molecules for miRNA binding. CeRNAs include lncRNA, circRNA and mRNA [35]. An interesting study found that, in three autophagy-related databases, *SLC7A11* gene expression was significantly decreased in drug-resistant ovarian cancer tissues and in two paclitaxel-resistant cell lines (HeyA8-R and SKOV3-R) compared to the paclitaxel-sensitive ovarian cancer tissues and cell lines (HeyA8 and SKOV3). Interestingly, the overexpression of SLC7A11 significantly increased the paclitaxel sensitivity of HeyA8-R cells, inhibited colony formation and induced apoptosis. Furthermore, low SLC7A11 expression was correlated with poor overall survival, progression-free survival and post-progression survival in ovarian cancer patients. Mechanistically, SLC7A11 modulated cell autophagy via a competing endogenous RNA (ceRNA) that interacts with the autophagy genes syntaxin 17 (*STX17*), RAS-associated binding protein 33 B (*RAB33B*) and ultraviolent (UV) irradiation resistance-associated gene (*UVRAG*). In fact, SLC7A11 was strongly and positively co-expressed with these three genes in drug-sensitive and -resistant ovarian cancer tissues. The authors also found that SLC7A11 induced the expression of autophagy genes, such as *LC3*, *Atg16L1* and *Atg7*. Moreover, the expression of these three autophagy genes was further increased when the cells were treated with paclitaxel. Thus, SLC7A11 can modulate autophagy via ceRNA interactions with the autophagy genes *STX17*, *RAB33B* and *UVRAG* in ovarian cancer [36].

Thus, SLC7A11 expression is inconsistent in paclitaxel- and cisplatin-resistant ovarian cancer since SLC7A11 expression is high in cisplatin-resistant but low in paclitaxel-resistant ovarian cancer, suggesting that SLC7A11 plays distinct roles in these two cases of chemotherapy resistance, possibly due to its modulation of different signaling pathways.

L-Alanosine (an amino acid analogue drug) and geldanamycin are two antibiotics with anticancer activity [37,38]. However, their effects can be modulated by SLC7A11 expression. In fact, an interesting study found that the inhibition of the $X_{c}^{-}$ transport system via the silencing of SLC7A11 expression (with siRNA) in SKOV3 ovarian cancer cells decreased sensitivity to L-alanosine, supporting the hypothesis that L-alanosine is an SLC7A11 substrate. In addition, SLC7A11 silencing increased geldanamycin efficiency, possibly due to the lowered intracellular glutathione levels. Thus, SLC7A11 regulates the cellular uptake of L-alanosine but confers resistance to geldanamycin by supplying cystine for glutathione maintenance. Therefore, an evaluation of SLC7A11 expression could be a predictor of a cellular response to L-alanosine and glutathione-mediated resistance to geldanamycin, suggesting that SLC7A11 could be a target in multi-drug chemotherapy [39].

Looking at the studies discussed in this section, it is possible to highlight the contrasting results reported in SLC7A11 expression in chemotherapy-resistant tissues and cell lines. In fact, two studies [31,33] reported an increased expression of SLC7A11, while two different studies [32,36] reported a decreased expression of SLC7A11 in chemotherapy-resistant ovarian cancer tissues and cell lines. These contrasting results could be due to the different techniques used to analyze SLC7A11 expression (protein or mRNA). Thus, further studies are necessary to evaluate the expression of SLC7A11 (possibly at both the mRNA and protein levels) in chemotherapy-resistant tissues and cell lines. 

## 3. SLC7A11 Modulation by Natural and Synthetic Compounds in Ovarian Cancer

Natural and synthetic compounds are widely used to treat several cancerous and noncancerous diseases [34,40,41,42,43,44,45,46]. Both these types of compounds can be used alone or in combination with chemotherapy to improve treatment outcomes or counteract the side effects of chemo- and radiotherapy in several types of cancers, including ovarian cancer. 

Agrimonolide is a natural compound isolated from Agrimonia pilosa Ledeb, a traditional Chinese medicinal herb with important anti-inflammatory, anticancer and antioxidant activities [47,48,49,50]. It has been reported that agrimonolide significantly inhibited the proliferation, migration and invasion of A2780 and SKOV-3 cells and also promoted apoptosis in these cells. Moreover, agrimonolide induced ferroptosis, resulting in the downregulation of sterol CoA desaturase (SCD1), SLC7A11 and GPX4 expression and an increase in ROS, total iron and Fe^2+^ levels [51]. SCD1 is a lipid-regulating enzyme that converts saturated fatty acids to ∆9-monounsaturated fatty acids [52]. SCD1 is highly expressed in several cancer types and modulates cancer cell initiation, represses apoptosis and promotes cancer cell proliferation [52]. In addition, SCD1 modulates ferroptosis in ovarian cancer [53]. Interestingly, SCD1 overexpression in A2780 and SKOV-3 cells significantly increased SLC7A11 and GPX4 protein expression. Thus, agrimonolide acts as an apoptosis- and ferroptosis-inducing agent in ovarian cancer cells and induces ferroptosis through the modulation of the SCD1/SLC7A11/GPX4 axis [51].

Cancer stem cells (CSCs) are cancer cells that are characterized by a high capacity for self-renewal and can be found in several liquid and solid cancers, thereby contributing to the onset, progression, recurrence and metastasis of these cancers [54]. The authors of an interesting study determined whether the ferroptosis pathway could be involved in the metabolism of fructose in ovarian CSCs by evaluating the expression of ferritin heavy chain subunits (FTH), which are subunits of an intracellular iron storage protein that contributes to the ferroptosis process [55,56], and SLC7A11, which acts as a negative regulator of ferroptosis [23]. These authors found that the treatment of spheroids comprising A2780 and SKOV3 CSCs with fructose significantly decreased SLC7A11 expression, while the increased expression of FTH resulted in an enrichment of the ferroptosis pathway (demonstrated via KEGG functional analysis). Therefore, fructose may be associated with ferroptosis and may modulate SLC7A11 expression in ovarian CSCs [57].

Eriodictyol is a flavonoid abundantly present in plants, fruits and vegetables with important anti-inflammatory, antitumor, cardioprotective and hepatoprotective effects [58,59]. The nuclear factor erythroid 2-related factor 2 (NRF2) is a key transcription factor involved in the antioxidant response [41,60]. It is known that NRF2 plays a key role in carcinogenesis, promoting tumor cell proliferation, invasion and chemoresistance in several types of cancers [43,61,62,63]. It has been reported that eriodictyol can regulate NRF2 expression and ferroptosis [64,65]. An interesting study evaluated the effects of eriodictyol on CaoV3 and A2780 ovarian cancer cells. It was found that eriodictyol treatment suppressed cell viability and induced apoptosis in both cell lines. Moreover, eriodictyol treatment significantly increased the Fe^2+^ content and ROS production and significantly decreased SLC7A11 and GPX4 protein expression. Finally, eriodictyol downregulated NRF2 expression in tumor tissues in mice xenograft models. Thus, eriodictyol regulated ferroptosis, ROS levels and cell viability and modulated NRF2 signaling in ovarian cancer [66]. These data are very important and are in agreement with two important studies [67,68] reporting that SLC7A11 is a prognostic ferroptosis-related gene in ovarian cancer. In fact, both these studies showed that low expression of SLC7A11 was associated with a poor overall survival in ovarian cancer patients [67,68]. Thus, the ferroptosis-related gene signature could predict overall survival in ovarian cancer patients and improve therapeutic treatments with a more personalized therapy.

Poly (ADP-ribose) polymerases (PARP) are a family of proteins involved in a variety of cellular processes, such as DNA repair, DNA methylation and programmed cell death [69,70,71]. Pharmacologic inhibition of PARP is the primary therapeutic strategy for BReast CAncer gene (*BRCA*) mutant ovarian cancer, but most patients carry wild-type *BRCA1/2* and show no clinical benefits from PARP inhibitors [72,73]. The *TP53* gene encodes the p53 tumor suppressor protein and is the most frequently mutated gene in human cancer, leading to the survival of cancer cells, ineffective therapeutic responses and unfavorable prognoses [74]. Importantly, it has been reported that p53 can repress SLC7A11 expression, which favors ferroptosis [75]. An interesting study found that ferroptosis is involved in the efficacy of olaparib, a PARP inhibitor. In fact, pharmacological inhibition of PARP by olaparib in HEY and A2780 ovarian cancer cells (which carry wild-type *BRCA1/2* [76]) significantly downregulated the expression of SLC7A11 and GPX4 and increased p53 expression, which promoted ferroptosis and DNA damage and led to cell apoptosis. Furthermore, it has been hypothesized that p53 could transcriptionally repress SLC7A11 expression, promoting ferroptosis in response to ROS-mediated stress (due to olaparib). This hypothesis was demonstrated by treating the p53-deficient ovarian cancer cell line SKOV3 with olaparib. In fact, olaparib reduced PARP1 protein expression without affecting SLC7A11 protein levels. The effect of p53 on SLC7A11 expression was further validated by deleting *p53* in wild-type *p53*-expressing HEY cells using the CRISPR/Cas9 system. According to the authors’ results, olaparib significantly downregulated SLC7A11 expression in control cells carrying the p53 protein but not in p53 knockout cells. Thus, olaparib represses SLC7A11 transcription primarily through the upregulation of p53 in ovarian cancer cells. Thus, combined treatment with a PARP inhibitor and a ferroptosis inducer could significantly improve the treatment of BRCA-proficient ovarian cancer [77]. 

Lidocaine is a local anesthetic drug with antimicrobial, antiarrhythmic and anti-inflammatory properties [78,79,80]. However, increasing evidence shows that lidocaine could interfere in the development of several cancers, including ovarian cancer [81]. An interesting study found that lidocaine can increase ferroptosis and thereby repress ovarian cancer progression. In fact, lidocaine treatment induced the accumulation of Fe^2+^ and increased ROS levels, which suppressed the proliferation, invasion and migration of SKOV-3 ovarian cancer cells and also promoted apoptosis in these cells. Mechanistically, the authors found that lidocaine treatment significantly downregulated SLC7A11 expression by enhancing miR-382-5p levels. In fact, the overexpression of miR-382-5p decreased SLC7A11 expression. The authors also found that the inhibition of miR-382-5p blocked lidocaine-induced ferroptosis in SKOV-3 cells. Thus, lidocaine can promote ferroptosis by modulating the miR-382-5p/SLC7A11 axis in ovarian cancer cells [82].

Multidrug resistance (MDR) is the primary cause of the failure of chemotherapeutic treatments and the low survival rates of ovarian cancer patients [83]. ABCB1 (also known as P-glycoprotein or MDR1), is a transmembrane protein involved in drug transport across the plasma membrane. ABCB1 plays a key role in cancer, and its overexpression reduces the accumulation of intracellular chemotherapeutics, leading to chemoresistance against several antineoplastic agents [84,85,86,87,88,89]. In fact, it has been proved that an increased expression of ABCB1 is involved in the occurrence of MDR in ovarian cancers treated with taxane drugs (e.g., Paclitaxel) [88,90,91,92]. Thus, the inhibition of ABCB1 may restore the sensitivity of ABCB1-substrate chemotherapeutic agents. Erastin is a ferroptosis inducer that inhibits SLC7A11, preventing cystine import and GSH depletion [93]. An interesting study found that cotreatment with erastin and docetaxel significantly decreased cell viability, promoted cell apoptosis and induced cell cycle arrest at the G2/M stage in docetaxel-resistant ovarian cancer (A2780/Taxol) cells (which overexpress the ABCB1 protein). Mechanistically, erastin binds ABCB1 and antagonizes its drug-efflux function, increasing the amount of intracellular docetaxel. In addition, the inhibition of SLC7A11 by erastin decreases the intracellular GSH content, further improving docetaxel efficiency. Thus, erastin could reverse ABCB1-mediated docetaxel resistance in ovarian cancer and improve chemotherapy efficiency in these patients [94].

The studies discussed in this section are summarized in Table 1 and clearly show that natural and synthetic compounds can efficiently regulate SLC7A11 expression in ovarian cancer cells. 

## 4. Intracellular Modulators of SLC7A11 in Ovarian Cancer

Many chemotherapeutic agents (e.g., platinum-derived drugs) rely on the induction of oxidative stress, which leads to cell damage and cancer cell death [95,96]. For this reason, targeting redox regulation in cancer cells is a promising strategy to overcome drug resistance. The transcription factor erythroblastosis virus E26 oncogene homolog 1 (Ets-1) is a transcription factor associated with chemotherapeutic resistance in ovarian cancer cells since an increased expression of Ets-1 leads to a decreased sensitivity to cisplatin treatment [97]. It has been found that the overexpression of Ets-1 in the human ovarian carcinoma cell line 2008 decreased intracellular ROS and increased intracellular GSH, GPX antioxidant activity, and SLC7A11 expression and activity. Under basal conditions, the inhibition of SLC7A11 by sulfasalazine decreased GPX activity in Ets-1 overexpressing cells. However, under oxidative stress, the intracellular GSH levels decreased significantly in correlation with increased Ets-1 expression following sulfasalazine treatment. Thus, Ets-1 regulates the enhanced SLC7A11 activity under oxidative stress [98].

CCAAT enhancer binding protein gamma (CEBPG) is a member of the C/EBP family of transcription factors, which is involved in cell differentiation and proliferation. CEBPG is an important regulator in tumor development and is highly expressed in cancer cells [99]. An interesting study found that CEBPG expression was increased in ovarian cancer tissues compared to normal ovarian tissues and was also associated with a poor prognosis. The knockdown of CEBPG inhibited the proliferation and invasion of A2780 and Hey ovarian cancer cells, promoting ferroptosis. Interestingly, *CEBPG* knockdown in A2780 and Hey ovarian cancer cells significantly decreased GPX4 and SLC7A11 expression. Moreover, the authors found that CEBPG could inhibit ferroptosis and promote the transcription of the *SLC7A11* gene by binding to its promoter region. Thus, CEBPG favors cancer progression by inhibiting ferroptosis through an increase in SLC7A11 expression [100].

AT-rich interacting domain containing protein 1A (ARID1A; also known as BAF250a and SMARCF1) is a component of the SWItch/sucrose non-fermentable (SWI/SNF) chromatin-remodeling complex, which makes chromatin accessible for transcription factor binding factor, allowing gene transcription [101,102]. *ARID1A* is frequently mutated in several cancers [103], including ovarian cancer, where *ARID1A* mutations/deletions are found in up to 80% of clear cell ovarian cancers (CCC), 40% of endometroid ovarian cancers and 30% of mucinous ovarian cancers. However, no *ARID1A* mutations/deletions have been reported in high-grade serous ovarian cancer [104]. An interesting study found that ARID1A-deficient cancer cells are specifically vulnerable to the inhibition of the glutamate-cysteine ligase synthetase catalytic subunit (GCLC), an important enzyme for GSH synthesis. In fact, the inhibition of GCLC significantly decreased GSH in ARID1A-deficient TOV21G ovarian cancer cells, leading to apoptosis (due to increased ROS levels). Interestingly, the authors found that the vulnerability of ARID1A-deficient cancer cells is caused by low basal GSH levels due to a decreased expression of SLC7A11. Importantly, the authors demonstrated that the overexpression of ARID1A in TOV21G cells significantly increased SLC7A11 protein expression. Thus, ARID1A-deficient ovarian cancers may be susceptible to drugs targeting GCLC since their efficiency is enhanced by a low SLC7A11 expression that keeps GSH content at low levels [105].

SNAI2 (also known as Slug) is a zinc-finger transcription factor that plays a key role in cancer onset, progression and metastasis, and an increased expression has been reported in several cancers [106], including ovarian cancer [107,108]. Jin and colleagues reported that an increased SNAI2 expression and ferroptosis were observed in SKOV3, A2780 and CAOV3 ovarian cancer cells compared to normal human ovarian surface epithelial (OSE) cells. *SNAI2* knockdown decreased cell migration and invasion and promoted cell apoptosis and ferroptosis in SKOV3 cells. Interestingly, the authors found that SNAI2 directly binds to the promoter of the *SLC7A11* gene and decreases its expression. SLC7A11 expression was also decreased after erastin treatment. Thus, SNAI2 can inhibit ferroptosis by blocking SLC7A11 expression and can therefore promote ovarian cancer progression [109].

The 3-hydroxy-3-methylglutaryl reductase degradation (HRD1) is a E3 ubiquitin ligase that plays an essential role in the ubiquitination and dislocation of misfolded proteins [110]. However, HRD1 can also target normally folded proteins for degradation. HRD1 is also involved in cancer progression, favoring cancer cell proliferation and growth in several types of cancers [110]. An interesting study found that HRD1 expression was significantly low in ovarian cancer tissues, and the overexpression of HRD1 inhibited the proliferation of SKOV3 and A2780 ovarian cancer cells by promoting apoptosis and ferroptosis. The authors also demonstrated that HRD1 interacted with and promoted the degradation of SLC7A11. Thus, HRD1 inhibited tumor formation and promoted ferroptosis in ovarian cancer cells by enhancing SLC7A11 degradation [111].

Long non-coding RNAs (lncRNAs) are non-coding RNA sequences that sponge and sequester microRNAs (miRNAs). Both lncRNAs and miRNAs regulate important cell processes in cancerous and noncancerous diseases [112,113,114,115]. It has been reported that lncRNA ADAMTS9-AS1 plays a key role in the progression and development of several types of cancer, including lung [116], glioma [117], bladder [118], breast [119] and prostate [119]. An interesting study found that the expression of lncRNA ADAMTS9-AS1 was significantly increased in ES-2, OVCAR3, SKOV3 and CAOV-3 ovarian cancer cells compared to normal human ovarian surface epithelial (OSE) cells. Interestingly, knocking down lncRNA ADAMTS9-AS1 by transfecting OVCAR3 and CAOV3 cells with siRNAs significantly increased miRNA-587 and decreased SLC7A11 expression. Moreover, knocking down lncRNA ADAMTS9-AS1 inhibited the proliferation and migration of ovarian cancer cells by promoting ferroptosis. The same results were obtained when miRNA-587 was overexpressed. Thus, lncRNA ADAMTS9-AS1 can attenuate ferroptosis by targeting the miR-587/SLC7A11 axis in ovarian cancer cell lines, thereby providing a new therapeutic target for the treatment of these patients [120].

Another study proved that SLC7A11 expression can be regulated by the lncRNA As-SLC7A11 [121], which is an antisense lncRNA (a lncRNA transcribed from the opposite strand of genes) [122]. In this study, the authors found that the level of lncRNA As-SLC7A11 was significantly decreased in epithelial ovarian cancer (EOC) tissues compared to normal tissues. In addition, lncRNA As-SLC7A11 levels were decreased in OVCA433, OVCA429 and TOV112D ovarian cancer cell lines compared with normal human ovarian surface epithelial (OSE) cells. The silencing of lncRNA As-SLC7A11 expression enhanced the cell migration and invasion of OVCA433 and OVCA429 cells, while the overexpression of lncRNA As-SLC7A11 induced apoptosis and decreased the expression of the SLC7A11 protein, suggesting that lncRNA As-SLC7A11 regulates the expression of SLC7A11 in ovarian cancer cells [121].

In addition to the lncRNAs, miRNA can also be regulated by circular RNAs (circRNAs), which are single-stranded covalently closed RNA molecules that regulate gene expression by interacting with miRNA [123,124]. CircRNAs play key roles in tumorigenesis and chemoresistance [123,124]. Qin and colleagues reported that cisplatin-resistant SKOV3/DDP and A2780/DDP cells show an increased expression of SLC7A11, GPX4 and circSnx12 compared to the cisplatin-sensitive SKOV3 and A2780 cells. Interestingly, the silencing of circSnx12 in SKOV3/DDP and A2780/DDP cells increased the expression of miR-194-5p, while decreased SLC7A11, GPX4 and GSH levels increased cisplatin sensitivity and ferroptosis. Moreover, miR-194-5p overexpression significantly decreased SLC7A11 expression. Thus, circSnx12 regulates ferroptosis and chemoresistance via the miR-194-5p/SLC7A11 pathway by sponging miR-194-5p [31].

The studies discussed in this section are summarized in Table 2 and show that SLC7A11 expression can also be efficiently modulated by cellular modulators, such as non-coding RNAs, Ets-1, CEBPG, ARID1A, SNAI2 and HRD1.

## 5. Conclusions

Ovarian cancer is among the most lethal gynecologic cancers and is characterized by high mortality and relapse rates due to late diagnosis and the frequent development of chemoresistance. Looking at the studies discussed in this review, it was revealed that SLC7A11 expression is significantly higher in ovarian cancer compared to normal tissues, but its expression in chemotherapy-resistant ovarian cancer tissues and cell lines is still a matter of debate since there are contrasting results (see Section 2), possibly due to the different techniques used to quantify SLC7A11 expression (protein or mRNA). The studies that evaluated SCL7A11-mediated cisplatin resistance suggest that this chemoresistance could be due to the high GSH content (due to the high expression of SCL7A11) in the cells that were studied. In fact, GSH decreases the efficiency of the ROS produced in response to cisplatin treatment, thus inhibiting ferroptosis (a ROS-mediated process).

Interestingly, the studies discussed in this review also showed that SLC7A11 expression can be downregulated by natural (agrimonolide, fructose and eriodictyol) and synthetic (olaparib, lidocaine and erastin) compounds (see Table 1), suggesting a potential use of these compounds in ovarian cancer treatment. It deserves to be mentioned that the function of the SLC7A11 protein can also be inhibited by several inhibitors or antagonists, such as sulfasalazine [125], HG106 [125], imidazole ketone erastin (IKE) [126] and sorafenib [127]. 

The problem of how to treat elderly patients who frequently have chronic medical illnesses will become more and more relevant in the next few years. At present, elderly cancer patients are not treated in the same manner as their younger counterparts not only for age-related organ failure but also for concomitant diseases. Thus, natural compounds could represent tolerable treatments for those patients whose comorbidities prevent them from undergoing radical surgery, which is the standard of care for this pathology [1]. In addition, important cellular modulators, such as non-coding RNAs, Ets-1, CEBPG, ARID1A, SNAI2 and HRD1 (see Table 2), could significantly regulate SLC7A11 expression in ovarian cancer cells, suggesting that these modulators could be potential therapeutic targets in ovarian cancer patients.

**Figure 2:**
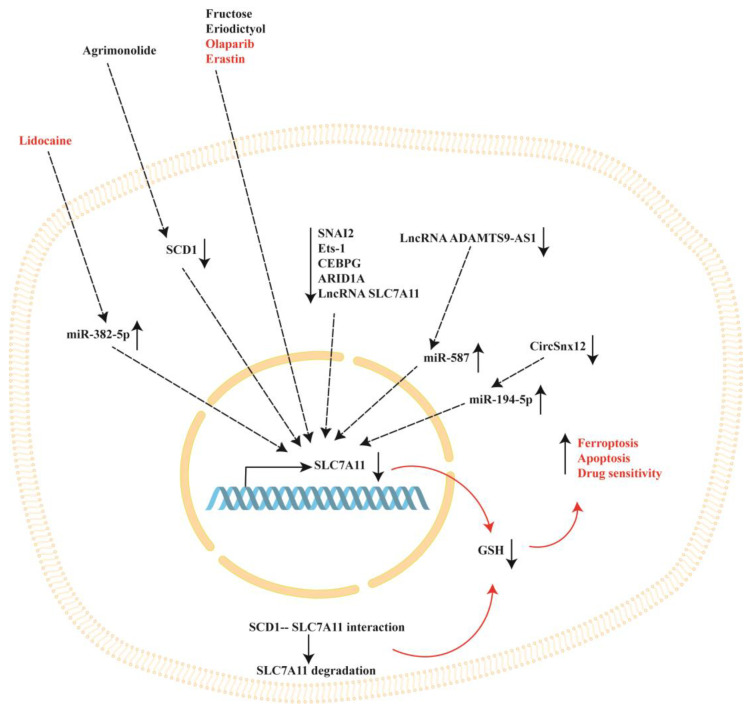
Representation of SLC7A11 modulation in ovarian cancer cells. SLC7A11 expression can be regulated by natural (in black outside the cell) and synthetic (in red outside the cell) compounds. In addition, SLC7A11 expression can be regulated by cellular modulators, such as miR-382-5p, miR-587, miR-194-5p, circSnx12, lncRNA ADAMTS9-AS1, lncRNA SLC7A11, sterol CoA desaturase (SCD1), SNAI2, erythroblastosis virus E26 oncogene homolog 1 (Ets-1), CCAAT enhancer binding protein gamma (CEBPG) and AT-rich interacting domain containing protein 1A (ARID1A). Decreased expression of SLC7A11 causes a decrease in intracellular glutathione (GSH) levels, which favors ferroptosis, apoptosis and drug sensitivity.

A schematic representation of SLC7A11 modulation in ovarian cancer is reported in Figure 2.

In conclusion, SLC7A11 could be a potential therapeutic target in ovarian cancer since it plays a key role in improving cancer cells’ response to chemotherapeutic agents in both sensitive and resistant ovarian cancer cells. Thus, specific clinical trials assessing the effects of SLC7A11 modulation (e.g., with natural or synthetic compounds) in ovarian cancer patients are needed to improve the outcomes of this disease. 

## Figures and Tables

**Table 1:** SLC7A11 modulation by natural and synthetic compounds in ovarian cancer.

Modulator	Model	Result	**Ref.**
Agrimonolide 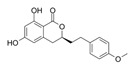	A2780 and SKOV-3 cells	Agrimonolide inhibited proliferation, migration, and invasion and promoted apoptosis. Agrimonolide induced ferroptosis, downregulated SCD1, SLC7A11 and GPX4 expression and increased ROS levels, total iron levels and Fe^2+^. SCD1 overexpression increased SLC7A11 and GPX4 protein expression. Agrimonolide acts as an apoptosis- and ferroptosis-inducing agent, inducing ferroptosis through the modulation of the SCD1/ SLC7A11/ GPX4 axis.	[51]
Fructose 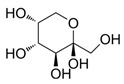	A2780 and SKOV3 CSCs in spheroids	Fructose treatment decreased SLC7A11 expression while increasing FTH expression, resulting in an enrichment of the ferroptosis pathway (demonstrated using KEGG functional analysis).	[57]
Eriodictyol 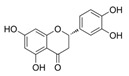	CaoV3 A2780	Eriodictyol suppressed cell viability and induced cell apoptosis. Eriodictyol increased the Fe^2+^ content and ROS production while reducing SLC7A11 and GPX4. Eriodictyol downregulated NRF2 expression in tumor tissues in mice xenograft models.	[66]
Olaparib 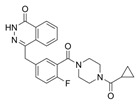	HEY and A2780 ovarian cancer cells (which carry wild-type BRCA1/2) and p53-deficient ovarian cancer cell line SKOV3	Olaparib downregulated SLC7A1 and GPX4 and increased p53 expression, promoting ferroptosis and DNA damage in HEY and A2780 ovarian cancer cells and leading to apoptosis. Treatment of SKOV3 cells with olaparib reduced PARP1 expression without affecting SLC7A11 protein levels. In addition, olaparib downregulated SLC7A11 expression in HEY control cells carrying the p53 protein but not in *p53* knockout cells. Olaparib represses SLC7A11 transcription through the upregulation of p53 expression.	[77]
Lidocaine 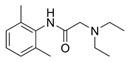	SKOV-3 ovarian cancer cells	Lidocaine induced the accumulation of Fe^2+^ and increased ROS levels, which suppressed proliferation, invasion and migration and promoted apoptosis. Lidocaine downregulated SLC7A11 expression by enhancing miR-382-5p levels. The overexpression of miR-382-5p decreased SLC7A11 expression, while the inhibition of miR-382-5p blocked lidocaine-induced ferroptosis in SKOV-3 cells. Lidocaine promotes ferroptosis by modulating the miR-382-5p/SLC7A11 axis.	[82]
Erastin 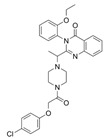	Docetaxel-resistant (A2780/Taxol) and -sensitive (A2780) ovarian cancer cells	Erastin and docetaxel cotreatment decreased cell viability, promoted cell apoptosis, and induced cell cycle arrest at the G2/M stage in A2780/Taxol cells (which overexpress the ABCB1 protein). Erastin binds ABCB1 and antagonizes its drug-efflux function, increasing the amount of intracellular docetaxel. The inhibition of SLC7A11 by erastin decreases the intracellular GSH content, further improving docetaxel efficiency.	[94]

**Table 2:** Cellular modulators of SLC7A11 expression in ovarian cancer.

Modulator	Model	Result	Reference
Ets-1	2008	The overexpression of Ets-1 decreased intracellular ROS and increased intracellular GSH, GPX antioxidant activity and SLC7A11 expression and activity. Under oxidative stress, the intracellular GSH levels decreased significantly in correlation with increased Ets-1 expression following sulfasalazine (a SLC7A11 inhibitor) treatment. Ets-1 regulates the enhanced SLC7A11 activity under oxidative stress.	[98]
CEBPG	A2780 and Hey ovarian cancer cells	The knockdown of CEBPG decreased GPX4 and SLC7A11 expression, inhibited cancer cell proliferation and invasion and promoted ferroptosis. CEBPG inhibited ferroptosis and promoted the transcription of the *SLC7A11* gene by binding its promoter region.	[100]
ARID1A	TOV21G ovarian cancer cells	ARID1A-deficient cells showed a decreased expression of SLC7A11 and are more sensitive to GSH inhibition treatments. The overexpression of ARID1A significantly increased SLC7A11 protein expression.	[105]
SNAI2	SKOV3, A2780 and CAOV3 ovarian cancer cells	SNAI2 knockdown decreased cell migration and invasion and promoted cell apoptosis and ferroptosis. SNAI2 directly binds to the promoter of the *SLC7A11* gene, decreasing its expression. SLC7A11 expression was also decreased after erastin treatment.	[109]
HRD1	SKOV3 and A2780 ovarian cancer cells	The overexpression of HRD1 inhibited proliferation and promoted apoptosis and ferroptosis. HRD1 interacted with SLC7A11 and promoted its degradation.	[111]
LncRNA ADAMTS9-AS1	ES-2, OVCAR3, SKOV3, CAOV-3 and normal human ovarian surface epithelial (OSE) cell line	LncRNA ADAMTS9-AS1 expression was increased in cancer cells. The knockdown of lncRNA ADAMTS9-AS1 in cancer cells inhibited cellular proliferation and migration by promoting ferroptosis, and miRNA-587 increased, while SLC7A11 expression decreased. The same results were obtained when miRNA-587 was overexpressed. LncRNA ADAMTS9-AS1 attenuates ferroptosis by targeting the miR-587/SLC7A11 axis.	[120]
LncRNA As-SLC7A11	OVCA433, OVCA429, TOV112D ovarian cancer cell lines and normal human ovarian surface epithelial (OSE) cell line	LncRNA As-SLC7A11 levels were decreased in ovarian cancer cell lines compared to normal OSE cells. The silencing of lncRNA As-SLC7A11 enhanced ovarian cancer cell migration and invasion, while its overexpression induced apoptosis and decreased SLC7A11 protein expression.	[121]
CircSnx12	Cisplatin-resistant SKOV3/DDP and A2780/DDP cells, and cisplatin-sensitive SKOV3 and A2780 cells.	SKOV3/DDP and A2780/DDP cells showed increased expression of SLC7A11, GPX4 and circSnx12 compared to the cisplatin-sensitive cells. The silencing of circSnx12 in SKOV3/DDP and A2780/DDP cells increased the expression of miR-194-5p and decreased SLC7A11, GPX4 and GSH levels, thus increasing cisplatin sensitivity and ferroptosis. MiR-194-5p overexpression decreased SLC7A11 expression. CircSnx12 regulates ferroptosis and chemoresistance via the miR-194-5p/SLC7A11 pathway by sponging miR-194-5p.	[31]

## Data Availability

Not applicable.
